# Shannon and Renyi Entropies to Classify Effects of Mild Traumatic Brain Injury on Postural Sway

**DOI:** 10.1371/journal.pone.0024446

**Published:** 2011-09-09

**Authors:** Jianbo Gao, Jing Hu, Thomas Buckley, Keith White, Chris Hass

**Affiliations:** 1 PMB Intelligence LLC, West Lafayette, Indiana, United States of America; 2 Affymetrix, Inc., Santa Clara, California, United States of America; 3 Graduate Athletic Training Program, Georgia Southern University, Statesboro, Georgia, United States of America; 4 Department of Psychology, University of Florida, Gainesville, Florida, United States of America; 5 Department of Applied Physiology and Kinesiology, University of Florida, Gainesville, Florida, United States of America; University of Maribor, Slovenia

## Abstract

**Background:**

Mild Traumatic Brain Injury (mTBI) has been identified as a major public and military health concern both in the United States and worldwide. Characterizing the effects of mTBI on postural sway could be an important tool for assessing recovery from the injury.

**Methodology/Principal Findings:**

We assess postural sway by motion of the center of pressure (COP). Methods for data reduction include calculation of area of COP and fractal analysis of COP motion time courses. We found that fractal scaling appears applicable to sway power above about 0.5 Hz, thus fractal characterization is only quantifying the secondary effects (a small fraction of total power) in the sway time series, and is not effective in quantifying long-term effects of mTBI on postural sway. We also found that the area of COP sensitively depends on the length of data series over which the COP is obtained. These weaknesses motivated us to use instead Shannon and Renyi entropies to assess postural instability following mTBI. These entropy measures have a number of appealing properties, including capacity for determination of the optimal length of the time series for analysis and a new interpretation of the area of COP.

**Conclusions:**

Entropy analysis can readily detect postural instability in athletes at least 10 days post-concussion so that it appears promising as a sensitive measure of effects of mTBI on postural sway.

**Availability:**

The programs for analyses may be obtained from the authors.

## Introduction

Traumatic Brain Injury (TBI) occurs when a direct or indirect blow to the head results in neuropathologic changes. In the United States, TBI represents a major medical concern that costs nearly $60 billion in direct and indirect expenses annually [1]–[3]. Most of these injuries are classified as mild TBI (mTBI) [1], with an estimated 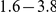 million injuries occurring in the United States annually as a result of sport participation [4]. The estimate likely represents underreporting: in one study [5], more than 50% of high school athletes failed to report their injuries to medical personnel. The acute and long term effects of mTBI also are prominent medical concerns for the armed services, as 15% of soldiers surveyed have experienced head trauma causing loss of consciousness or altered mental status while serving in Iraq [6].

Current clinical assessment protocols have demonstrated strong sensitivity (89–96%) in acutely identifying the presence of mTBI [7]; however, no such instruments have successfully identified the recovery process or when an individual has “healed”. Attempts to elucidate recovery following concussion has been limited both by the unique aspects of the individual's mTBI and by limitations in the assessment tools. Indeed, structural imaging techniques (MRI, CT) are of limited value beyond classifying the injury as “mild” as the pathophysiology of mTBI is generally considered to be a functional disorder [8]. Traditional clinical assessment techniques such as graded symptom checklists (GSC), standard assessment of concussion (SAC) cognitive assessment, balance error scoring system (BESS), and neuropsychological (NP) tests have 1) demonstrated inconsistent ability to acutely identify the presence of a concussion, 2) have not been validated to identify recovery from mTBI, and 3) are substantially limited by a practice effect [9]–[11]. As premature return to participate presents an acute risk of the potentially fatal second impact syndrome [12] as well as elevated risk of repeat concussion and associated potential long term sequele including mild cognitive impairment [13], earlier onset of Alzheimer disease [13], chronic traumatic encephalopathy [14] and amyotrophic lateral sclerosis [15]. Thus, the development of a sensitive method to identify healing following mTBI represents a pressing need in the neurological and sports medicine communities.

The assessment of postural control provides an interesting means of identifying concussion-related neurophysiological abnormality. It is one of several recommended tools for determining readiness to resume competitive activity among athletes [16]. Previous research has suggested that athletes with postural instability after concussion return to their baseline levels of postural steadiness performance within about 3 days often despite still being symptomatic [17]–[20]. This suggests that either (a) neurophysiological impairments affecting postural control are not necessarily a predictable consequence of injury, or (b) more sensitive analyses of postural instability may be required.

The methods currently used to assess postural control following mTBI may be one limiting factor in better classifying the severity of mTBI as well as elucidating the path to recovery from the injury. Traditional measures of postural sway based on variance of ground reaction forces, or their extremes observed within a given time window, are likely to be insensitive to the effects of the injury, since they are largely controlled by body weight. Fractal analysis of COP signals, while promising for identifying differences in postural stability between control and elderly subjects [21]–[24], has not been shown whether it can characterize the instability of postural control following mTBI. While the notion of virtual time-to-contact (VTC) is able to indicate effects of TBI to about 30 days post injury [25], the mechanism for such a capability is unclear. Approximate entropy (ApEn), a metric from nonlinear dynamics theory, seems to offer more insight into classifiable characteristics of the postural control system [26], [27]. The analyses are however, based on fairly short data sets with limited parameter combinations. This raises an important question regarding the adequacy of those analyses.

To gain deeper insights into the dynamics of postural instability following mTBI, in this work, we ask three fundamental questions: (1) What is an optimal length for the data that would be adequate for assessing postural control following mTBI? (2) How effectively can fractal analysis assess postural control following mTBI? (3) Can a general information theoretic approach based on Shannon and Renyi entropies be developed such that it can assess postural instability as well as shed light on the effectiveness of other methods for analyzing COP signals?

## Methods

### 1. Experimental procedure and data

Ten varsity intercollegiate student athletes with mTBI or with recent diagnosis of concussion participated in this study. Three of the ten subjects had a history of a prior concussion (0.7+1.3, R = 0–3) and, utilizing the Cantu revised evidence based grading scale [28], eight of the ten subjects were classified as grade II concussions while two were classified as grade III. Further, 50% of the subjects reported post traumatic amnesia and 40% of the subjects reported loss of consciousness, however only one was lasted longer than a few seconds. All subjects denied current and past history of balance, neurological, metabolic, or vestibular disorders. All subjects provided written informed consent prior to participating as approved either by the University of Florida Institutional Review Board, for studies conducted by Dr. Hass, or by the Georgia Southern University Institutional Review Board, for studies conducted by Dr. Buckley. Analyses of these existing data, which are reported in the present paper, were also disclosed to the University of Florida and Georgia Southern University Institutional Review Boards, and to the US Army Research Office Human Research Protections Office, and were determined to be exempt.

**Figure 1:**
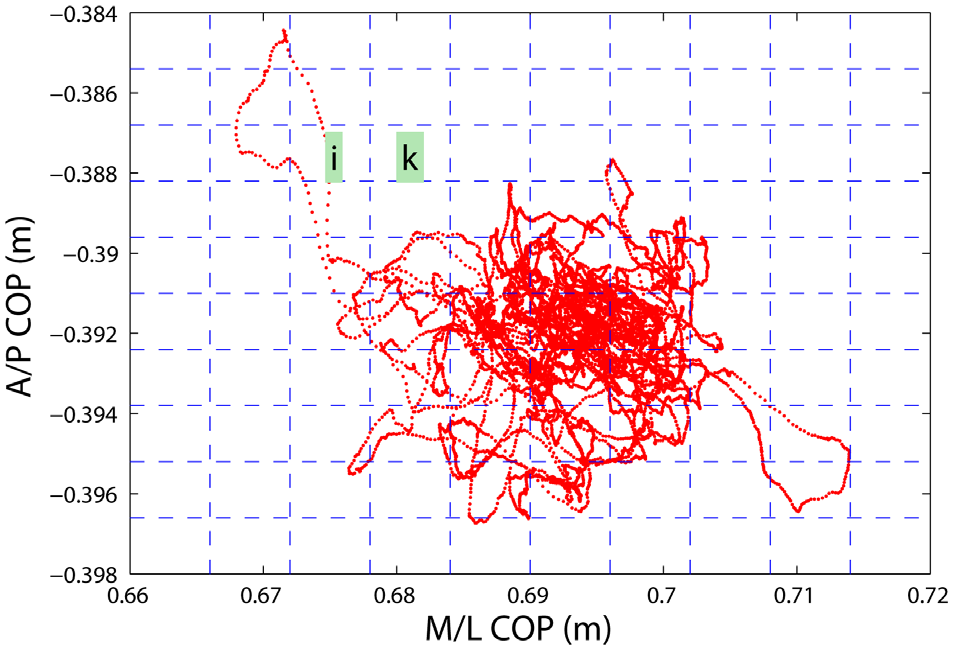
COP trajectory for a subject on day 6 after concussion.

Potential subjects were identified by the athletic training staff and the concussion diagnosis was confirmed by both the treating certified athletic trainer and the team physician. On the day following the concussion, Day 1, the experimental procedures were initiated. The subject reported to the biomechanics laboratory for testing each day until they were cleared to return to participation in accordance with university medical policies, seven days symptom free with progressive exertion (11.8+2.5 days). The last day of testing was the day the subject was cleared for return to full activity in their sport. One subject suffered a second concussion during the recovery from his initial concussion and did not return to participation that season. Upon arrival at the biomechanics laboratory, each subject performed one trial of static stance for 2 minutes. The subject was instructed to stand barefoot on the force platform and to remain as stationary as possible for the duration of the experiment. The trial was initiated and concluded with a verbal cue from the investigator. Ground reaction forces (A/P, M/L, and vertical) and center of pressure (A/P and M/L) data were collected from a single force platform (model OR-6, AMTI, Watertown, MA, USA) at 1000 Hz, where A/P and M/L denote Anterior/Posterior and Medial/Lateral balance, respectively. An example of a COP trajectory is shown in Fig. 1 for a subject on day 6 after concussion.

### 2. Calculation of area of COP trajectory

The area of the COP trajectory is a popular metric for characterizing postural sway. Consider a COP trajectory such as shown in Fig. 1. To compute the area the trajectory has traced out, one can partition a 2-dimensional plane that encompasses the trajectory into unit areas, and sum up all the non-empty unit areas covered by the COP trajectory.

### 3. Detrended fluctuation analysis

Detrended fluctuation analysis (DFA) [29]–[31] characterizes the second order statistic – the correlation, in a time series. It can automatically remove certain trends or nonstationarity contained in the data under study. When applying DFA, one works on a random-walk-type process, and expects the process to have a power-law decaying spectral density. Denote the COP data by 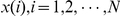. The random-walk-type process 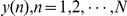 can be obtained by first removing the mean value 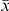 and then forming partial summation,
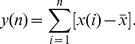
(1)DFA works as follows. First, one divides the time series into 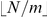 non-overlapping segments (where the notation 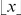 denotes the largest integer that is not greater than 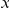), each containing 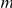 points; then one calculates the local trend in each segment to be the ordinate of a linear least-squares fit for the random walk in that segment, and computes the “detrended walk”, denoted by 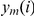, as the difference between the original walk 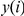 and the local trend; finally, one examines if the following scaling behavior (i.e., fractal property) holds or not:
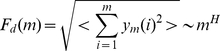
(2)where the angle brackets denote ensemble average of all the segments. The parameter 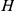 is often called the Hurst parameter [32]. When the scaling law described by Eq. (2) holds, the process under investigation is said to be a fractal process. The autocorrelation for the “increment” process, defined as 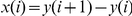, decays as a power-law,
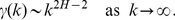
(3)When 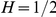, the process is called memoryless or short range dependent. The most well-known example is the Brownian motion (Bm) process. In nature and in man-made systems, often a process is characterized by an 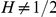. Prototypical models for such processes are fractional Brownian motion (fBm) processes. When 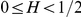, the process is said to have “anti-persistent” correlations [32]. For 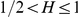, the process has “persistent” correlations, or long memory properties [32]. The latter is justified by noticing that
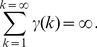
(4)In practice, quite often power-law relations are only valid for a finite range of 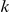. Unfortunately, some researchers try to estimate the 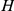 parameter (or other scaling exponents such as the fractal dimension) by some optimization procedure without being concerned about the scaling region.

### 4. Calculation of Shannon and Renyi entropies

As we have discussed, to compute the area of the COP, one can count the number of non-empty grids/boxes covered by the COP trajectory. The idea can straightforwardly be extended to calculate Shannon and Renyi entropies, by the following procedure.

Assume a trajectory has visited 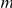 unit areas, with the 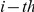 unit area being visited by 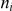 times. This is schematically shown in Fig. 1. Note that an empty unit area, such as that denoted by 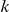 in Fig. 1 is irrelevant. Let the trajectory be 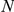 points long. Then the probability 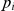 that the 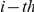 unit area being visited is 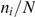. The Shannon entropy is defined by
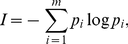
(5)where the unit for 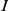 is a bit or baud corresponding to base 2 or 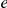 in the logarithm. Without loss of generality, we shall choose base 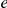.

Renyi entropy is a generalization of Shannon entropy. It is defined by
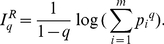
(6)

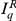
 has a number of interesting properties:

- When 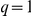, 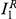 is the Shannon entropy: 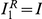.

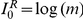
 is the topological entropy, which is just the logarithm of the area traced out by a sway trajectory. Therefore, the case of 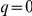 is equivalent to the logarithm of the area.
- If 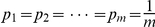, then for all real valued 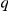, 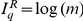.
- In the case of unequal probability, 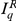 is a nonincreasing function of 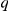. In particular, if we denote
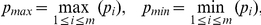
then
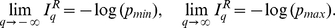

It is clear that so far as the unit areas are not visited with equal probability, the Renyi entropy provides a better and more comprehensive characterization than the area metric. In particular, we can envision two potential advantages:

- Unit areas that are visited very rarely have very small probability. Sometimes, they could correspond to outliers or “errors”. Their effect can be mitigated by making 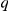 larger.
- If instead unit areas with small probability are to be “weighed” more, then one can simply make 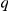 smaller.

## Results

### 1. Optimal data length for analysis

**Figure 2:**
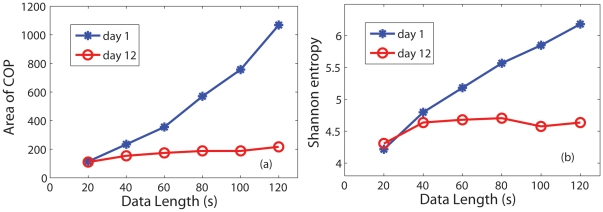
Variation of area and Shannon entropy with data length. (a) area and (b) Shannon entropy vs. data length for a subject on day 1 and day 12 after concussion.

A standard balance test may last only 20 s [26]. Would such short data be adequate for calculating nonlinear entropy metrics from postural sway data? To see the problem, we have first calculated a popular metric for characterizing postural sway, the area of the COP. Specifically, we have calculated the area of COP based on the first 20 s, first 40 s, first 60 s, first 80 s, first 100 s, and all 120 s data. The variation of the area vs. such data length for one subject is plotted in Fig. 2(a). We observe that on day 1 after concussion, the area increases with the data length rapidly. Actually, the growth rate is faster than linear! From other subjects' data, we have always observed that on day 1 after concussion, the area increases with data length at least linearly, and sometimes even exponentially.

The result shown in Fig. 2(a) compels us to ask: i) How long should the data be to make proper conclusions? Note that so far as area is the metric, no easy answer can be given, since area is a strictly non-decreasing function of data length. Note also that the work by Cavanaugh et al. [26] using approximate entropy was based on 20 s data. They commented “Paradoxically, the range of COP displacement after injury (approximately 4 cm) was less than at preseason (approximately 5 cm), suggesting that postural stability had improved, rather than become more impaired, after injury.” Their puzzling observation could just be due to the shortness of data they analyzed, noticing that the increase of area with data length is much slower on day 12 than on day 1 – in other words, postural instability would not be fully revealed by short data right after concussion. ii) How should comparisons be made among different injured subjects before traditional clinical measures such as GSC, SAC, or BESS scores return to baseline? This is a harder question to answer. Especially, depending on the severity of concussion, one subject's day 1 behavior could be similar to another subject's day 2, day 3, or even other day's behavior.

While a clean, definitive answer to both questions might be hard to obtain, later in Sec. 3, we shall develop an information theoretic approach so that we can gain important insights into these issues.

### 2. Fractal analysis for assessing postural instability

Numerous work has shown that gait dynamics can be modeled by 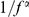 processes, where 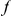 is frequency and 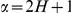, where 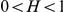 is called the Hurst parameter and characterizes the correlation structure of the process: depending on whether 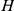 is smaller than, equal to, or larger than 1/2, the process is said to have anti-persistent, short-range, or persistent long-range correlations [29], [30]. Fractal analysis is also very promising for identifying differences in postural stability between control and elderly subjects [21]–[24]. This motivates us to ask whether the key parameter, 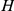, from fractal analysis, can be used to indicate postural instability after concussion.

**Figure 3:**
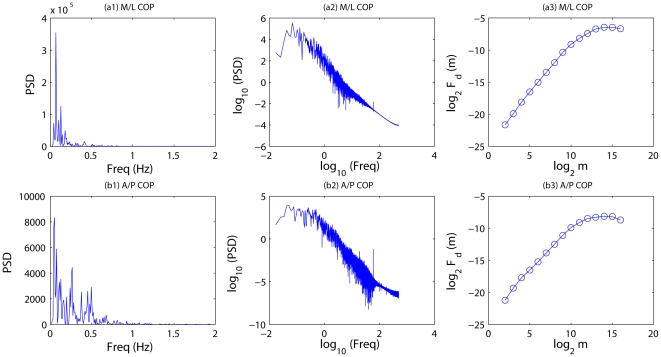
Power spectral density (PSD) and DFA results for COP of subject CW04 on day 1. First column: PSD in linear scale; 2nd column: PSD in log-log scale; observe the linear relation on high frequency end. 3rd column: DFA results; indeed, there is very good linear (or scaling) relation.

**Figure 4:**
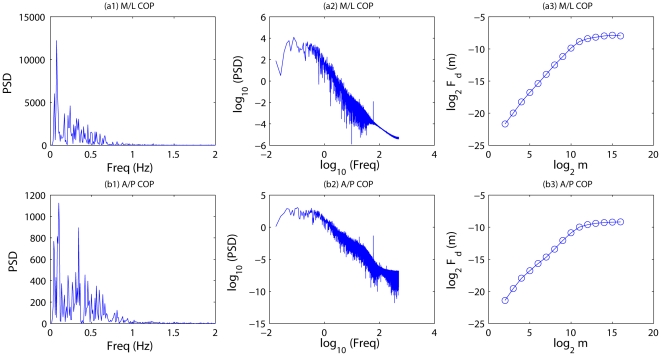
Same as **Fig. 3** except for day 10.

To answer the above important question, we have systematically examined the frequency contents of sway data. Figs. 3–4 show the power spectral density (PSD) for COP on day 1 and day 10, where the 1st column is always for PSD in linear scale; the 2nd column for PSD is in log-log scale. At the first sight, the 2nd column is very interesting: we clearly observe a linear line in log-log scale for both A/P and M/L COP data, indicating that COP data may be modeled by 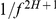 processes.

To further confirm the scaling law of the COP data, we apply a more sophisticated method, DFA, which is a more reliable method for fractal analysis [29], [30]. The DFA curves for the COP data of one subject for day 1 and day 10 are shown in Figs. 3–4 as the 3rd column, where the quantities are plotted also in log-log scale – when the curve is linear, it means the process is a fractal process, with 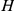 given by the slope of the linear curve. Indeed, the curves are quite linear. In fact, the 2nd and 3rd columns correspond well: the 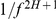 scaling is from about 1 Hz up for COP, amounting to 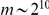 samples in the DFA curve. Further, the 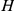 estimated from PSD and DFA curves are consistent. Unfortunately, 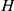 here is always greater than 1, and is not effective in indicating postural instability after concussion. This compels us to have a serious 2nd thought about the frequency contents of the data.

It turns out significant understanding can be found from the 1st column of Figs. 3–4: only low frequencies have appreciable power; the frequency range where fractal scaling is observed basically has negligible power. The 1st columns of Figs. 3–4 actually have indicated how long the sway data have to be for a meaningful analysis. For example, the frequency with the largest power in Figs. 3(a1,b1) is around 0.1 Hz. Thus, if one only uses 20 s data, as Cavanaugh et al. did [26], one basically only observes about 4 cycles of variations, if one assumes the frequency to be cut at 0.2 Hz. Since data here are not really periodic, but rather random, little can be inferred from data as short as 20 s.

### 3. Shannon and Renyi entropies for assessing postural instability

We now check how Shannon entropy varies with data length. The result is shown in Fig. 2(b). We observe two interesting features: (1) Shannon entropy for data measured on day 1 after concussion keeps increasing with data length, while that on day 12 reaches saturation when data length is 40 s; with longer data length, it fluctuates slightly. The latter feature reflects that the trajectory visits different unit boxes with un-equal probability. (2) While overall, Shannon entropy for day 1 is much larger than that for day 12, when data length is as short as 20 s, the opposite is actually the case. Recall that a similar behavior has also been observed with approximate entropy with 20 s data right after concussion [26]. We now see that associating the value of approximate entropy with the complexity of postural sway based on such short data may not be justified.

**Figure 5:**
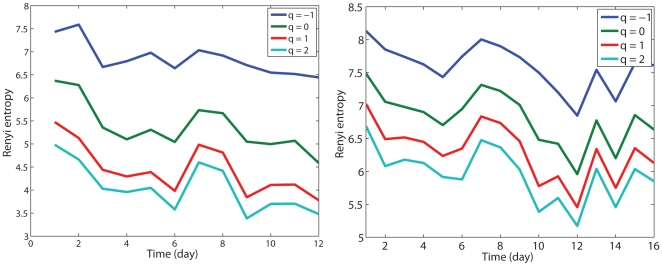
Temporal variations of Renyi entropies for 2 subjects. The 2nd subject (right) had two concussions: day 7 was the 1st day of the 2nd concussion.

**Figure 6:**
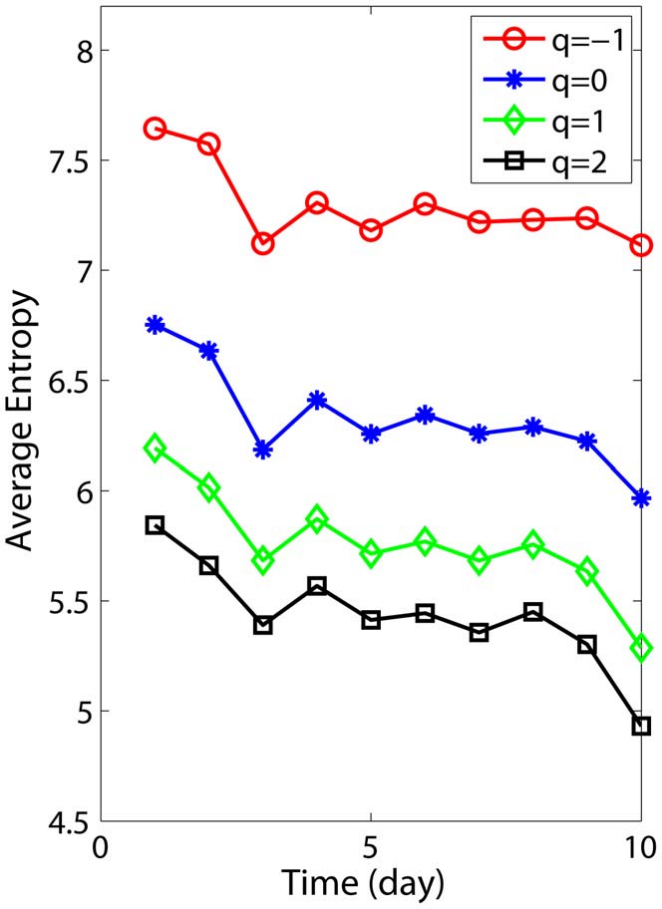
Temporal variations of Renyi entropies averaged over 10 subjects.

The above discussions make it clear that we have to use all 120 s of the data, in order to reliably assess the effects of postural instability after concussion. The variations of entropies with the number of days after concussion are shown in Fig. 5 for two subjects. When such curves are averaged over the 10 subjects (up to day 10, which is the last day for some subjects), we obtain Fig. 6. While we identify that the general trend for the variation of the Renyi entropies is decreasing after concussion, suggesting recovery from concussion, we emphasize that the actual variation of the Renyi entropies is not simply monotonic, but quite complicated. This implies that the subjects might not have fully recovered from concussion-induced postural instability, even after a fairly long period of time (such as 1–2 weeks). This also means that these entropy measures can effectively indicate postural instability long after concussion has occurred. Note that there is a RTP protocol that lets concussed student athletes return to sports activity only 4–5 days after injury [33]. Our analysis indicates that such a clinical practice may be too aggressive.

### 4. Dealing with nonstationarity

Postural sway data are notoriously nonstationary. One motivation that the standard practice only collects 20–30 s data is to suppress nonstationarity. Now that we have shown that 20–30 s data are too short, the issue of nonstationarity becomes more acute. Therefore, an emerging challenge would be to properly deal with nonstationarity and remove noise from sway data, so that subsequent analysis is meaningful.

**Figure 7:**
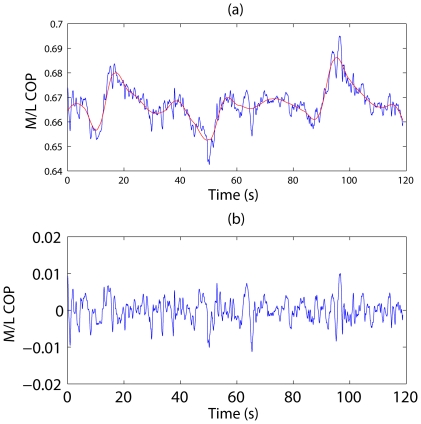
Detrending of M/L COP signal. **(a) M/L COP signal (blue) and the complicated trend (red) determined by adaptive detrending.** The difference between them is the stationary M/L COP signal shown in (b).

In this regard, a versatile adaptive algorithm for detrending, denoising, multiscale decomposition, and multifractal analysis recently developed by the authors may be very useful [34]–[37]. In fact, as far as denoising is concerned, the method is better than linear filters, wavelet and chaos-based approaches [36]. While we omit the details of the method here, we would like to show an example (Fig. 7) to illustrate the effectiveness of the method. Clearly, the long-term trend has been accurately determined.

It is important to note that the trend in Fig. 7(i.e., the red curve) may have its own significance; therefore, whether it should be filtered out or not depends on an understanding of the mechanism for the trend signal. Overall, we may conclude that nonstationarity associated with longer data will not pose a challenge in data analysis.

## Discussion

MTBI is a major public and military health concern. To help assess recovery from mTBI, in this paper, we have carefully examined the effects of COP data length on the computation of a popular metric, the area of COP. We have found that immediately following concussion, the area of COP data increases with data length at least linearly for data length up to 2 min, therefore, at least 2 min data is required in order to reliably quantify the effects of mTBI on postural instability. We have also examined the utility of fractal analysis for assessing postural instability, and found that fractal scaling appears applicable to sway power above about 0.5 Hz, thus fractal characterization is only quantifying the secondary effects (a small fraction of total power) in the sway time series, and not effective in indicating recovery following mTBI. More interestingly, we have developed an information theoretic approach to quantify postural instability, by defining Shannon and Renyi entropies from COP data. These entropy measures have a number of appealing properties, including capacity for determination of the optimal length of the time series for analysis and a new interpretation of the area of COP. Most importantly, entropy analysis can readily detect postural instability in athletes at least 10 days post-concussion so that it appears promising as a sensitive measure of effects of mTBI on postural sway.

We emphasize that our purpose here is to develop suitable concepts to effectively quantify postural instability. The data analyzed here may be considered minimal for verifying our concepts. In future, it will be very desirable that data collection can be more systematic, in the sense that normal, pre-concussion data can also be collected, together with post-concussion data long after the injury, such as one month after the injury. It will also be interesting to re-examine approximate entropy using longer data, to gain more insights into the findings of Cavanaugh et al. [26].
